# A Rare Case of Malignant Mesothelioma Presenting with Systemic Lupus Erythematosus Seropositivity: A Case Report and Review of Literature

**DOI:** 10.7759/cureus.4092

**Published:** 2019-02-19

**Authors:** Amandeep Rakhra, Ahmed Munir, Ramya S Chilukuri, Joseph Nahas

**Affiliations:** 1 Internal Medicine, Creighton University, Omaha, USA; 2 Rheumatology, Creighton University, Omaha, USA

**Keywords:** systemic lupus erythematosus (sle), malignant mesothelioma

## Abstract

While malignant mesothelioma may initially present in a variety of ways, it is uncommon to present with systemic lupus erythematosus (SLE) seropositivity and thus obscuring its diagnosis. Our case involves a 75-year-old Caucasian male with a past medical history of essential hypertension, remote prostate cancer status post prostatectomy, and lifetime nontobacco use presenting with progressive shortness of breath over one month. After a negative cardiac assessment, a postcardiac catheterization chest X-ray (CXR) revealed a right-sided moderate-to-large pleural effusion that, on further workup, was found to be exudative. Effusion studies were negative for malignancy and bacterial growth. Recurrent accumulation of fluid after a thoracentesis one week prior prompted an autoimmune work up. Positive markers included antinuclear antibodies, anti-double stranded DNA antibodies, and anti-histone antibodies, while anti-Smith antibodies were negative. Although SLE was initially suspected based on serologies, no clinical signs or symptoms were present to fulfill the diagnosis criteria. A trial of oral prednisone resulted in decreased pleural effusion size with no further recurrence. Additional studies included a CT scan of the chest that showed pleural masses confirmed with biopsy to be epithelioid mesothelioma. Given the patient’s age and new diagnosis of malignant mesothelioma, we hypothesized that the presence of autoantibodies was likely false positives due to acquired autoantibodies with age, hyperactivity of the immune system from malignancy, and possible prior asbestos exposure.

## Introduction

Malignant mesothelioma is a relatively uncommon malignancy, with an annual incidence of 3000 cases in the USA [1]. Typically, it appears in those with asbestos exposure and history of tobacco use. It is rare for mesothelioma to have an association with a connective tissue disorder. There have been two reported cases in the literature describing the initial diagnosis of malignant mesothelioma with systemic lupus erythematosus (SLE) seropositivity; however, both met at least four criteria for a diagnosis of SLE. SLE is most commonly diagnosed in young, African American females aged 16-55. Incidence rates of SLE in the USA are 20-150 new cases per 10,000 each year [2]. Associations between malignancies and rheumatologic seropositivity have been studied, including the potential influence of occupational exposures; however, little is known about how and why. The presence of certain autoimmune antibodies has also been associated with certain malignancies without any underlying rheumatologic processes. Given the wide range of initial presentations of malignancies, it is important to keep a broad differential and recognize appropriate clinical contexts in order to make accurate diagnoses.

## Case presentation

**Figure 1:**
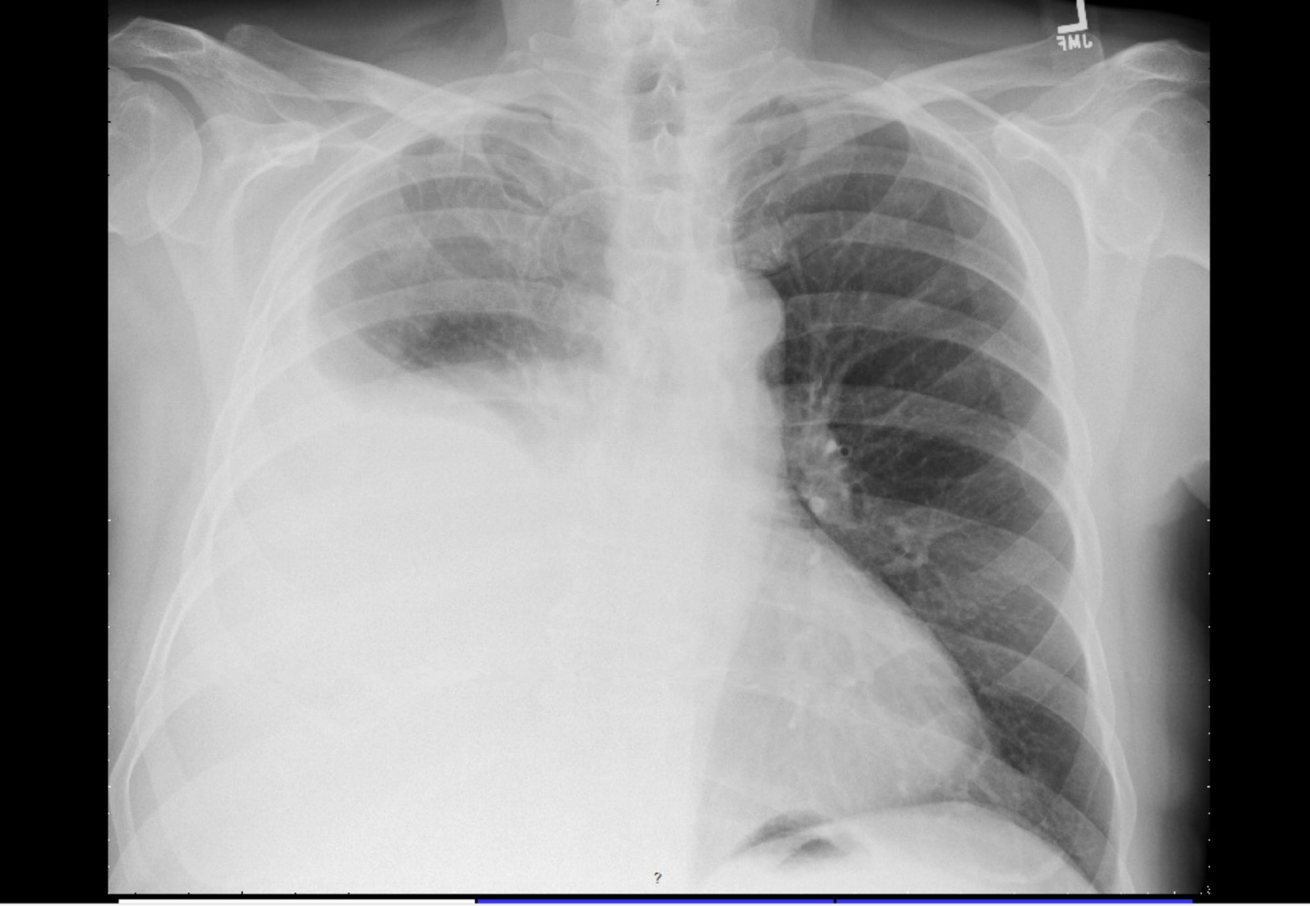
Chest X-ray demonstrating the right moderate-to-large pleural effusion.

A 75-year-old Caucasian male with a past medical history of essential hypertension, prostate cancer status post prostatectomy, and lifetime nonsmoker presented to his primary care provider with progressive shortness of breath and chest heaviness for one month. He denied systemic symptoms including weight loss, fevers, chills, or appetite loss. He reported ongoing productive cough with clear sputum. He was urgently referred to cardiology, in which an exercise stress test yielded ST-segment depression coinciding with anginal symptoms. Cardiac catheterization was performed and unremarkable for coronary disease. A post-catheterization chest X-ray (CXR) was significant for a right hemithorax with a moderate-to-large pleural effusion (Figure 1). He was then sent to pulmonology for a thoracentesis, with three liters of pleural fluid removed. Pleural fluid studies indicated an exudative effusion that was negative for both malignancy and bacterial growth. He initially reported improvement of his dyspnea, however, his symptoms reappeared after a few days. Recurrent accumulation of fluid evident on CXR one week later prompted an additional thoracentesis and further evaluation for secondary causes, including autoimmune-mediated processes.

Serology results included the presence of antinuclear antibodies (ANA), low-titer anti-double stranded DNA (anti-dsDNA) antibodies 15 IU/mL, and rheumatoid factor (RF) 16 IU/mL. Anti-histone antibodies (AHA) were moderately positive at 2.5 Units. Anti-Smith antibodies and anti-cyclic citrullinated peptide (anti-CCP) antibodies were absent. Both erythrocyte sedimentation rate (ESR) and C-reactive protein (CRP) were elevated at 52 mm/h and 32 mg/L, respectively. C3 and C4 complement levels and urinalysis with microscopy were normal. Table 1 includes laboratory results with their normal references ranges.

**Table 1:** Laboratory results with normal reference ranges.

	Values	Normal reference range		Values	Normal reference range
ANA, qualitative screen	Positive	-	AHA	2.5 Units	0.0-0.9 Units
Anti-dsDNA	15 IU/mL	0.0-4.0 IU/mL	ESR	52 mm/h	0-15 mm/h
Anti-Smith	Negative	-	CRP	32.70 mg/L	<=9.00 mg/L
RF	16 IU/mL	<=15 IU/mL	C3 Complement	156 mg/dL	90-180 mg/dL
Anti-CCP	<20 Units	<20 Units	C4 Complement	35 mg/dL	15-40 mg/dL

**Figure 2:**
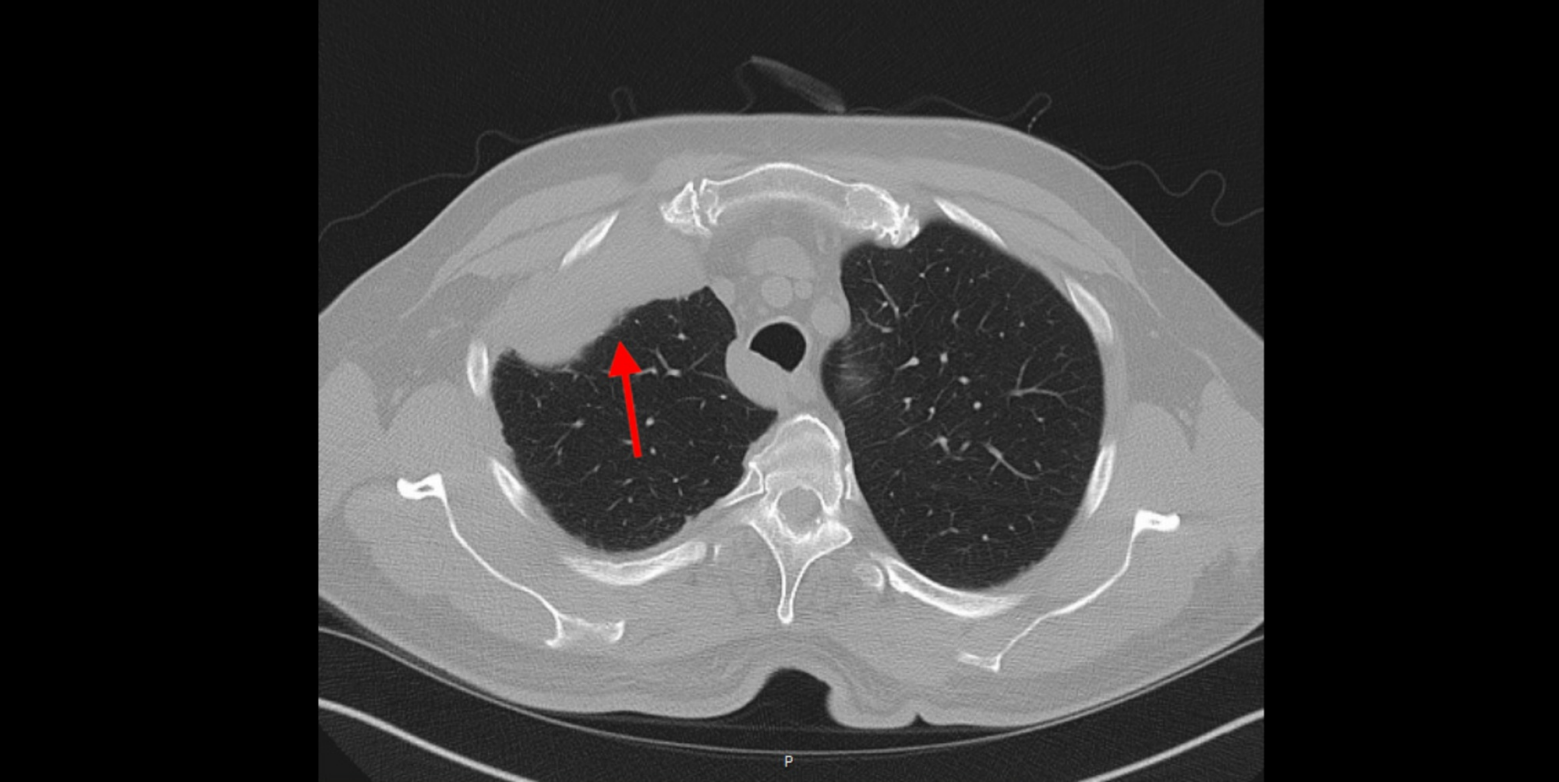
Transverse cross-section of CT Chest. Anterior pleural mass of the right upper lobe (red arrow).

**Figure 3:**
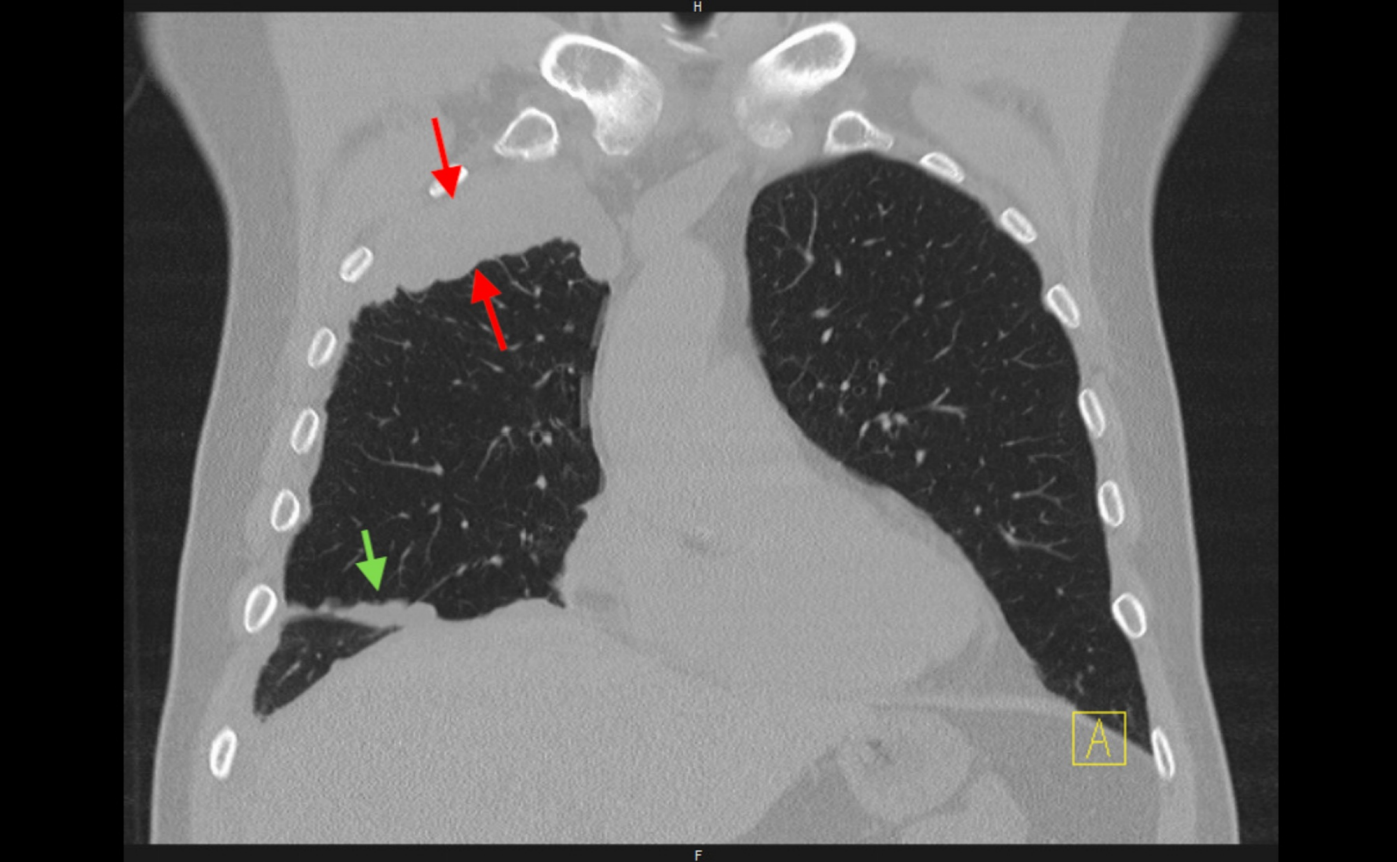
Coronal cross-section of CT Chest. Right upper lobe pleural mass (red arrows) with nodularity of the right oblique fissure (green arrow).

In the setting of positive ANA, anti-dsDNA, and AHA, the patient was referred to Rheumatology for possible SLE. The patient denied classic systemic symptoms associated with SLE, including arthralgias, joint swelling, skin rash, or Raynaud’s phenomenon. However, it was still believed that his pleural effusion was secondary to an autoimmune etiology. He was started on a trial of oral prednisone 30 mg daily for seven days. A repeat ultrasound one week later demonstrated a decrease in size of the pleural effusion. Further evaluation with a CT scan of the chest revealed multiple pleural masses, including a 7.8 cm x 2.4 cm lobulated pleural mass in the right upper lobe. Additionally, there was nodularity of the right mediastinal and diaphragmatic pleura, suggestive of possible pleural mesothelioma. The presence of enlarged cardiophrenic lymph nodes was indicative of potential metastatic disease (Figures 2-3).

**Figure 4:**
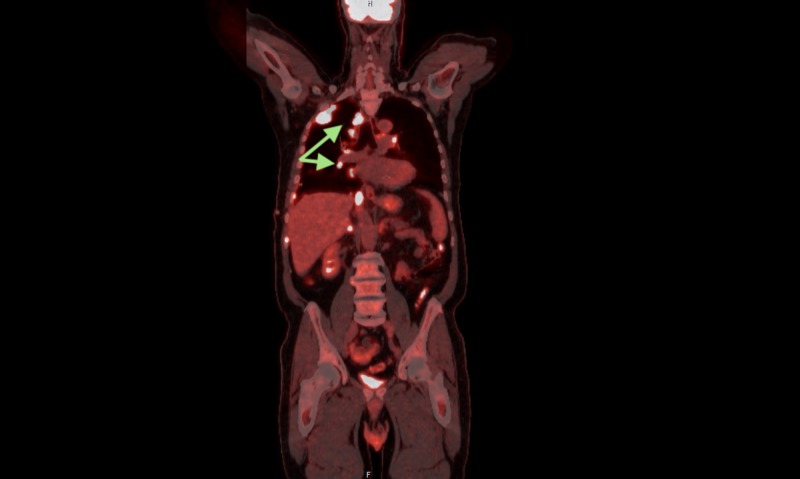
Coronal view, positron emission tomography (PET) scan. Hilar lymphadenopathy of right hemithorax (green arrows).

**Figure 5:**
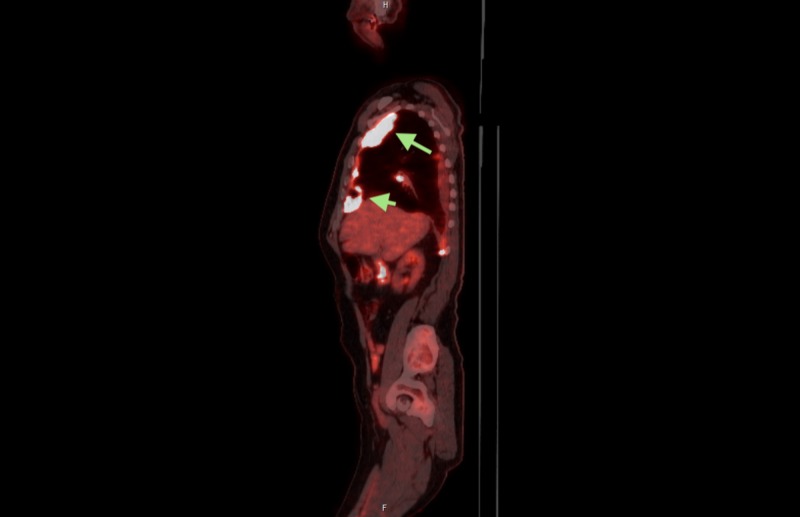
Sagittal view, PET scan. Evidence of pleural based bases of right hemithorax (green arrows).

A bronchoscopy with lung biopsy was consistent with epithelioid mesothelioma. A positron emission tomography (PET) scan revealed hypermetabolic activity corresponding with the lesions demonstrated on the CT scan of the chest (Figures 4-5). The patient was then referred to Hematology/Oncology where he underwent treatment for his newly diagnosed malignant mesothelioma. 

## Discussion

Here we have a case of malignant mesothelioma masquerading as a presumed SLE-mediated pleural effusion. Annual incidence of mesothelioma is approximately 3000 cases per year, with approximately 50% of cases secondary to asbestos exposures [1]. SLE is a well-known autoimmune disorder more commonly diagnosed in females aged 16-55 of African American descent, with reported 12%-18% of cases being diagnosed after the age of 50 [3]. The incidence of SLE in men has been estimated to be one in every 10 cases of diagnosed lupus [4]. SLE can affect multiple organ systems including the lungs. Pleurisy, either with or without pleural effusions, is the most common pulmonary manifestation in patients with SLE [5]. Approximately 50% of pleural effusions associated with SLE are bilateral and exudative in nature [6].

It is unclear why SLE is more common in women—theories of estrogen influence, hypogonadism, and androgen deficiencies have been previously disproven. There have been conflicting reports regarding differences in clinical manifestations between both sexes; however, men have been found to be diagnosed at a later age [4, 7]. A 2012 study published in The Journal of Rheumatology found that the majority of men with SLE reported symptom onset and diagnosis at the age of 30 or older. Furthermore, men were less likely to present with arthritis but have higher rates of renal complications and hemolytic anemia [7]. In 2001, a study involving male veterans with SLE consisted of 2144 subjects—the largest cohort of this population to be studied. It was suggested that men had a later age of symptom onset, with higher mortality at one year after diagnosis than females. This leads to the hypothesis that men with SLE endure a more complex clinical course than their female counterparts [8]. Still more needs to be studied regarding this demographic.

There have only been two reported cases in the literature of mesothelioma presenting with SLE seropositivity. Both cases were diagnosed in women aged 40-50 with mesothelioma of the pericardium. The first case was published in 1984 and discussed the importance of keeping a broad differential in the setting of low rheumatologic titers. At least seven criteria for SLE were met including positive ANA, serositis, and photosensitive malar rash. Despite treatment for SLE, she continued to deteriorate and further workup led to a diagnosis of mesothelioma [9]. This emphasized the importance of combining diagnostic criteria with the clinical presentation, with serologies acting as a guide to make the diagnosis. 

The second case, published over 30 years later in 2017, involved a patient diagnosed with a pericardial effusion thought to be secondary to SLE. After recurrent effusions despite appropriate therapy, CT scan of the chest showed thickening of the pericardial layer. Pericardiectomy and biopsy later confirmed the diagnosis of pericardial mesothelioma. Although a rare presentation. malignant mesothelioma may present with systemic autoimmune features, thus complicating its diagnosis. It is important to keep in mind that recurrent effusions without obvious etiologies should prompt additional investigation beyond rheumatologic conditions and into potential neoplasms [2].

Despite associating rheumatologic markers with only rheumatologic disorders, there has been evidence of their connections with other underlying pathologies. In 2010, a study published in Saudi Arabia evaluated patients with positive anti-dsDNA antibodies to explore potentially non SLE-related etiologies. Out of 212 patients with positive anti-dsDNA antibodies, 124 had SLE, 29 had other rheumatologic diseases (i.e. anti-phospholipid antibody syndrome, rheumatoid arthritis, etc.), and 11 with infections such as cellulitis or osteomyelitis. There were six patients noted to have an underlying malignancy including, but not limited to, lymphomas and cancers of the breast, lungs, and stomach. The occurrence of high anti-dsDNA antibody titers, specifically over 800 IU/mL, is more likely suggestive of rheumatologic processes [10]. Overall, this study concluded that the presence and level of anti-dsDNA antibodies can include multiple differentials. It was also mentioned that the use of enzyme-linked immunosorbent assay (ELISA) for anti-dsDNA antibody detection has been known to result in false positives [11], although specific false positive rates have not been established and may vary depending on individual laboratories. In some countries, a positive ELISA would prompt additional confirmation with Crithidia luciliae immunofluorescence testing (CLIFT) or radioimmunoassays [12].

Anti-histone antibodies were also mildly positive in our patient. Typically, anti-histone antibodies are associated with drug-induced lupus erythematosus (DILE). Common medications known to cause DILE include hydralazine, sulfasalazine, and procainamide. Our patient was not on any typical offending medications to suggest DILE. The presence of AHA can also be misleading. A study in Spain assessed a database with those who were AHA positive between 2000 and 2016. Out of 73 patients with positive AHA, zero developed DILE. On the other hand, approximately half of patients did develop another form of autoimmune disease, such as SLE or Sjogren’s syndrome. Coincidentally, it was noted that anti-dsDNA antibodies were the most commonly co-expressed rheumatologic marker in AHA positive patients [13]. It is also important to recognize multiple subtypes of histones. Each subtype can be linked to several pathologies, extending from autoimmune to malignancy [14]. This, however, has not been further studied when evaluating links between AHA positivity and cancer specifically.

Prior studies have also indicated drug-induced subcutaneous lupus erythematosus (SCLE) to be associated with positive AHA. Hydrochlorothiazide was one of the first medications attributed to such and, less common, angiotensin-converting-enzyme (ACE) inhibitors, specifically captopril [15]. Our patient had been taking a hydrochlorothiazide-lisinopril combination tablet for essential hypertension, yet he did not have any clinical signs or symptoms to suggest SCLE.

Systemic lupus erythematosus has been linked to certain malignancies over time, particularly Non-Hodgkin’s lymphoma, vulvar, liver, and lung cancers. Lymphomas tend to be more commonly associated with SLE, specifically in younger patients [16]. In addition, a connection between SCLE and malignancies has been recognized as early as 1982. The first case involved a 70-year-old male who presented with skin lesions and systemic symptoms suggestive of SCLE whilst diagnosed with lung adenocarcinoma. It was noticed that his cutaneous manifestations improved with the treatment of his cancer. Since then, multiple tumors in occurrence with autoimmune processes have been recognized, including breast and multiple gastrointestinal malignancies [15]. The connection is thought to be secondary to a mutational oncogene that is in close proximity to another gene responsible for immune-mediated processes [16]. Malignancy itself, however, triggers inflammation in the body and potentially results in autoantibody production [17]. 

A correlation between occupational exposures and autoantibody positivity has also been observed. Silica exposure can lead to the production of autoimmune antibodies via mechanisms not quite known, increasing the risk of rheumatologic conditions such as SLE, scleroderma, and rheumatoid arthritis (RA). Additionally, asbestos may potentially lead to autoantibody production: ANA, anti-dsDNA, and RF are a few that have been reported. RA is the most common systemic autoimmune disease associated with asbestos exposure [18]. Our patient initially denied exposure to both asbestos and silica; however, he later mentioned the possibility of asbestos exposure during his time in the military. This may have contributed to the elevation of these autoantibodies as well as his mesothelioma. 

Our patient was initially thought to have SLE as the cause of his recurrent pleural effusions given positive SLE serologies; however, he did not present with common clinical symptoms. He had elevation of multiple rheumatologic markers, including ANA, RF, low titers of anti-dsDNA antibodies, and moderately positive AHA. The presence of ANA and RF could be explained by the patient’s age as acquired autoantibodies alone. It would have been beneficial for a quantitative ANA titer, however, only a qualitative screen was completed making its presence nonspecific. With negative anti-CCP antibodies and the absence of arthralgia and synovitis, RA was a less likely etiology. Although anti-dsDNA is sensitive for SLE, they lack the specificity of anti-Smith antibodies, which were negative in our patient. Anti-dsDNA antibodies and AHA could potentially be considered false positives or attributed to malignancy. Additionally, we cannot exclude the possibility of his prior asbestos exposure contributing to his serology positivities. A distinct etiology for our patient’s positive AHA is still unclear; however, we believe it is more likely due to an overactive immunologic response rather than an underlying autoimmune disease. Complement levels were also normal in our patients but are typically low in SLE.

Although antibodies for SLE appear prior to symptom onset, this is likely not the case in our patient. Even so, it is unlikely for SLE to present in a male during his eighth decade of life. There are potentially multiple explanations for his positive serology, however, it is important to assess the entire clinical picture. One must also keep in mind that malignancies, including mesothelioma, may be also associated with positive SLE serology.

## Conclusions

Malignancies may be associated with autoantibody production due to an overwhelming immunologic response. Malignant mesothelioma has been associated with positive rheumatologic markers without any underlying rheumatologic disorders. Given the wide range of initial presentations of malignancies, it is important to keep a broad differential and recognize clinical contexts in order to make an accurate diagnosis for timely treatment.
